# Telehealth: An Important Player During the COVID-19 Pandemic

**DOI:** 10.31486/toj.20.0039

**Published:** 2020

**Authors:** Naureen Akber Ali, Adeel Khoja

**Affiliations:** ^1^ School of Nursing and Midwifery, Aga Khan University, Karachi, Pakistan; ^2^ Department of Medicine, Aga Khan University, Karachi, Pakistan; ^3^ Department of Cardiology, The University of Adelaide, Adelaide, Australia

## TO THE EDITOR

The novel coronavirus (COVID-19) has rapidly spread across national and international borders, causing severe illness in countries throughout the world and creating a serious public health crisis.^1,2^ The number of ill patients and those suspected to be infected has burdened hospitals and medical equipment supply chains.^3^ One way to mitigate some of the logistics problems created by the COVID-19 pandemic is to transition to telehealth services when possible.

Augmenting healthcare systems with telehealth services is feasible, and telemedicine gives practitioners a way to offer medical support to patients during this global pandemic.^3^ The US Centers for Disease Control and Prevention, several state public health agencies, many European Union countries, and China have modified existing rules and regulations to permit the increase in utilization of telehealth services and have promoted telehealth as part of the response to the novel coronavirus.^3-6^

Telehealth services can be provided through devices such as computers, smartphones, and tablets.^4^ Doctor-patient interaction can still occur despite social distancing and stay-at-home orders,^5^ and telehealth services eliminate the exposure risk inherent in an in-person clinic visit. Telehealth can be used to monitor patients recovering from COVID-19 after their discharge from the hospital.^7^ Telehealth also provides a way to reach populations that have low or no access to care.

Telehealth has also become important in the provision of mental health services,^4^ including the provision of services for clinical teams at high risk of mental distress because of exposure to infected patients. Such distress can cause burnout, stress, anxiety, and depression that can ultimately have a deleterious impact on a health system's capacity to deliver services during this time of crisis.^8,9^ In response to COVID-19, several telehealth mental health services were implemented in China, including counseling, supervision, training, and psychoeducation for frontline clinicians, patients who tested positive for COVID-19 and their families, and policemen and security guards.^4^

Transitioning to telehealth services when possible can also help conserve medical supplies.^3^

Public health agencies, governments, stakeholders, and policymakers must introduce this technology throughout the world, including the South Asia regions where only 0.7 to 2.8 critical care beds per 100,000 population are available and where diagnostic and critical care facilities are limited.^10,11^

Telehealth services are an efficient way to provide people with remote access to quality healthcare services without increasing the risk of transmitting infection. The COVID-19 pandemic may be the impetus for governments and regulatory agencies to adopt telehealth services and integrate them into existing health systems globally.
